# Conserved gene signalling and a derived patterning mechanism underlie the development of avian footpad scales

**DOI:** 10.1186/s13227-019-0130-9

**Published:** 2019-08-13

**Authors:** Rory L. Cooper, Victoria J. Lloyd, Nicolas Di-Poï, Alexander G. Fletcher, Paul M. Barrett, Gareth J. Fraser

**Affiliations:** 1 Department of Animal and Plant Sciences, University of Sheffield, Sheffield, UK; 2 Program in Developmental Biology, Institute of Biotechnology, University of Helsinki, Helsinki, Finland; 3 School of Mathematics and Statistics, University of Sheffield, Sheffield, UK; 4 Department of Earth Sciences, Natural History Museum, London, UK; 5 Department of Biology, University of Florida, Gainesville, USA

**Keywords:** Epithelial appendage, Placode, Reticulate scale, Chicken, Patterning, Reaction–diffusion

## Abstract

**Background:**

Vertebrates possess a diverse range of integumentary epithelial appendages, including scales, feathers and hair. These structures share extensive early developmental homology, as they mostly originate from a conserved anatomical placode. In the context of avian epithelial appendages, feathers and scutate scales are known to develop from an anatomical placode. However, our understanding of avian reticulate (footpad) scale development remains unclear.

**Results:**

Here, we demonstrate that reticulate scales develop from restricted circular domains of thickened epithelium, with localised conserved gene expression in both the epithelium and underlying mesenchyme. These domains constitute either anatomical placodes, or circular initiatory fields (comparable to the avian feather tract). Subsequent patterning of reticulate scales is consistent with reaction–diffusion (RD) simulation, whereby this primary domain subdivides into smaller secondary units, which produce individual scales. In contrast, the footpad scales of a squamate model (the bearded dragon, *Pogona vitticeps*) develop synchronously across the ventral footpad surface.

**Conclusions:**

Widely conserved gene signalling underlies the initial development of avian reticulate scales. However, their subsequent patterning is distinct from the footpad scale patterning of a squamate model, and the feather and scutate scale patterning of birds. Therefore, we suggest reticulate scales are a comparatively derived epithelial appendage, patterned through a modified RD system.

**Electronic supplementary material:**

The online version of this article (10.1186/s13227-019-0130-9) contains supplementary material, which is available to authorized users.

## Background

Integumentary epithelial appendages are a diverse group of organs that includes scales, feathers, teeth and hair [1]. These structures facilitate a broad range of functions, such as communication, protection, thermoregulation and locomotion [2–4]. Recent research has revealed they share developmental homology, as they mostly originate from a conserved epithelial placode, which develops within an initiatory field such as a feather tract [5–8]. This placode is characterised by conserved patterns of gene expression in the epithelium and underlying mesenchyme, as well as columnar basal epithelial cells which exhibit a reduced rate of proliferation [5, 9, 10]. The spatial distribution of these conserved placodes during development, and therefore the ultimate pattern of adult epithelial appendages, is important for facilitating their diverse functions.

Epithelial appendage patterning is thought to be controlled by a reaction–diffusion (RD) system, whereby interactions between differentially diffusing activatory and inhibitory morphogens give rise to autonomous pattern formation [11, 12]. Previous research has indicated that RD is of widespread importance during epithelial appendage patterning of species from a diverse range of taxonomic groups, from sharks to mammals [8, 13, 14]. RD mediates the spatial distribution of individual epithelial placodes, which subsequently undergo morphogenesis and differentiate into their final adult form.

However, further research has demonstrated that there are exceptions to this patterning mechanism. The head scales of crocodiles are not individual developmental units. Instead, they arise from the physical cracking of highly keratinised skin, presenting a stochastic patterning system distinct from RD [15]. Additionally, mechanosensory forces in the tissue are considered to be important for the initiation of follicle patterning in avian skin [16, 17]. This demonstrates that alternative processes contribute to the diversity of vertebrate epithelial appendage patterning.

**Fig. 1:**
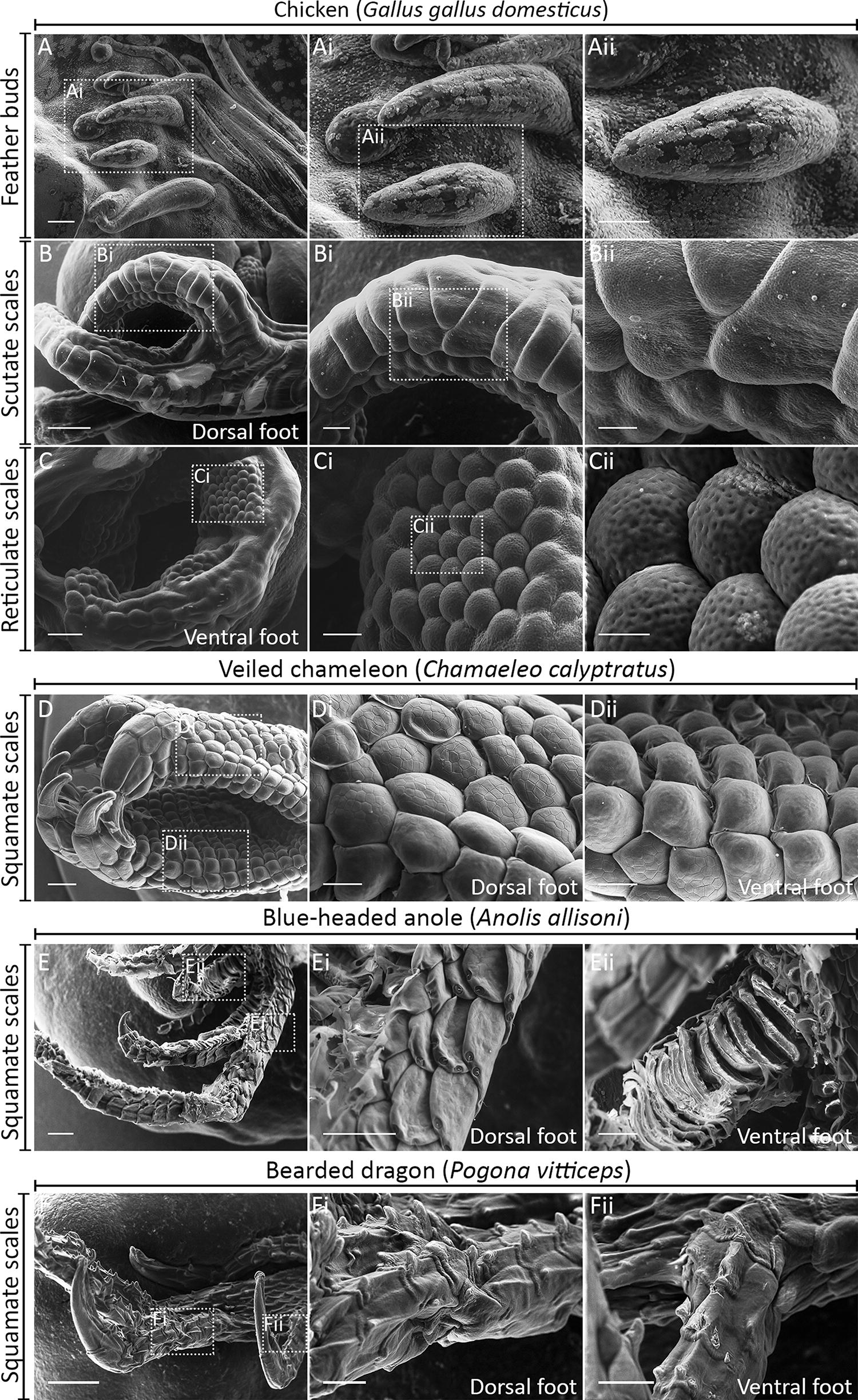
Morphological diversity of avian and reptilian integumentary appendages. Scanning electron microscopy (SEM) was used to examine the morphological characteristics of avian and reptilian appendage types. The E14 chicken embryo (*Gallus gallus*) possesses feathers (**A**), scutate scales on the metatarsal shank and dorsal foot surface (**B**), and reticulate scales on ventral foot surface (**C**). The hatchling veiled chameleon (*C. calyptratus*) possesses bilateral scales on the dorsal and ventral foot surface, which bare morphological similarity to reticulate scales (**D**). The hatchling blue-headed anole (*A. allisoni*) (**E**) and the E46 bearded dragon (*Pogona vitticeps*) (**F**) possess large overlapping scales, more similar to avian scutate scales. Scale bar lengths are: **A**, **Bi**, **Di**, **Dii**, **Ei**, **Eii**, **Fi**, **Fii** = 125 µm, **Ai**, **Aii** = 50 µm, **B**, **D**, **F** = 500 µm, **Bii**, **Ci** = 75 µm, **C**, **E** = 250 µm, **Cii** = 25 µm

The chicken embryo is an important model for studying epithelial appendage development and associated RD patterning [8]. Chickens possess a range of epithelial appendages, including feathers (of which there are several types, from filoplume to flight feathers [18]) and various scale types [19] (Fig. 1A–C). Overlapping scutate scales are found on the anterior metatarsal shank and the dorsal surface of the foot, whereas radially symmetrical reticulate scales are typically found on the ventral surface of the foot and digits (Fig. 1B–C) [20], presumably to provide cushioning and grip during locomotion.

There is uncertainty regarding the evolutionary relationships between different squamate and avian scale types [5, 21]. It has been hypothesised that squamate reptilian scales share more similarity with avian reticulate scales than avian scutate scales [22]. However, the identification of an anatomical placode in squamate scale development indicates that reticulate scales might be derived structures [5, 6, 23]. Reticulate scales may be distinct from other amniote epithelial appendages due to the apparent lack of individual epithelial placodes [6, 24]. A recent transcriptome sequencing (RNA-seq) analysis showed that gene expression during feather development is more similar to that of scutate scale development than expression during reticulate scale development [25]. One conclusion from this study suggested that reticulate scales are comparatively less derived than feathers and scutate scales, potentially representing a more primitive state. Separate research compared avian epithelial appendage development and proposed that scutate scales are secondarily derived from feathers [26]; however, this study did not examine reticulate scales.

Although feathers have provided a widely used model system for studying avian epithelial appendage development [8, 27], the development of reticulate scales has been largely unexplored at both cellular and molecular levels. Developmental studies exploring reticulate scales are absolutely necessary to improve our understanding of both the evolutionary relationships between different avian and squamate epithelial appendage types, and the evolution of avian-specific epithelial appendages.

Here, we examine the development of epithelial appendages in the chicken (*Gallus gallus*), focusing upon the patterning of reticulate scales. Using scanning electron microscopy (SEM), in situ hybridisation (ISH) and immunofluorescence, we ask whether the development of reticulate scales is underpinned by conserved gene signalling, known to be important throughout the development of other avian and squamate epithelial appendage types. Additionally, we investigate whether reticulate scale development follows a patterning mechanism consistent with RD simulation during their propagation throughout the footpad.

## Results

### Avian and squamate scales exhibit morphological diversity

**Fig. 2:**
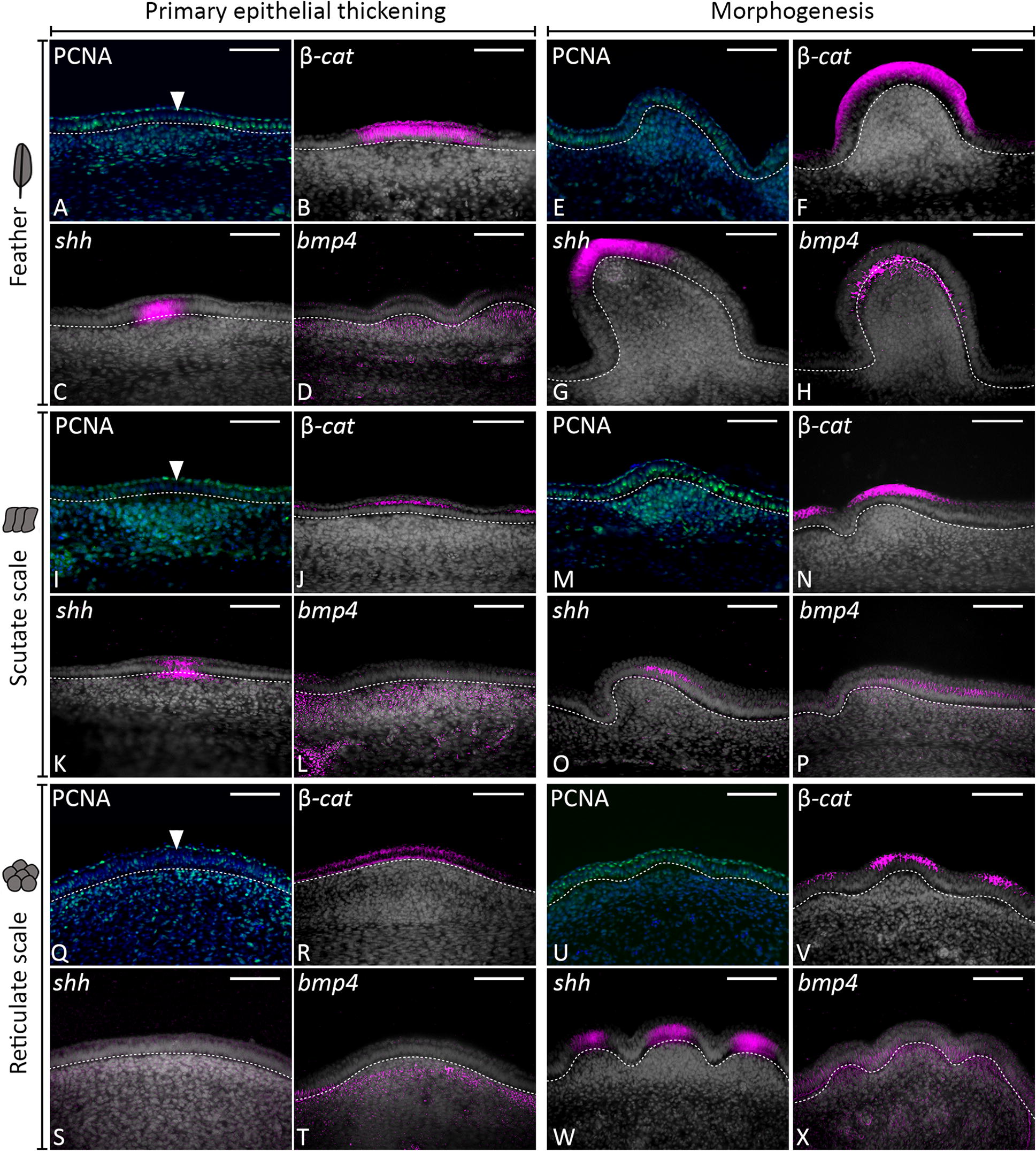
Conserved gene signalling underlies the development of feathers, scutate and reticulate scales. Vibratome sectioning of whole-mount ISH samples was done to examine tissue layer-specific expression of *β*-*cat*, *Shh* and *Bmp4* during development of avian epithelial appendages. Sections shown are false coloured, with DAPI in grey and gene expression in pink. Immunoreactivity of PCNA was also examined, with DAPI in blue and PCNA in green. PCNA immunoreactivity revealed columnar cells of the basal epithelium with reduced proliferation compared to surrounding cells during the primary epithelial thickening stage, for feathers, scutate and reticulate scales (**A**, **I**, **Q**) (white arrowheads). *β*-*cat* expression was localised to the epithelium during both the primary stage and morphogenesis of chick feather, scutate and reticulate scale development (**B**, **F**, **J**, **N**, **R**, **V**). Similarly, *Shh* expression was localised to the epithelium, although at the reticulate scale primary epithelial thickening stage, localised expression was not observed (**C**, **G**, **K**, **O**, **S**, **W**). Expression of *Bmp4* was mesenchymal during the primary stage and observed in both the epithelium and mesenchyme during morphogenesis (**D**, **H**, **L**, **P**, **T**, **X**). Overall, these results suggest avian appendage development is underpinned by conserved gene signalling. White dashed lines separate the basal epithelium from the mesenchyme. Scale bars are 75 µm in length

**Fig. 3:**
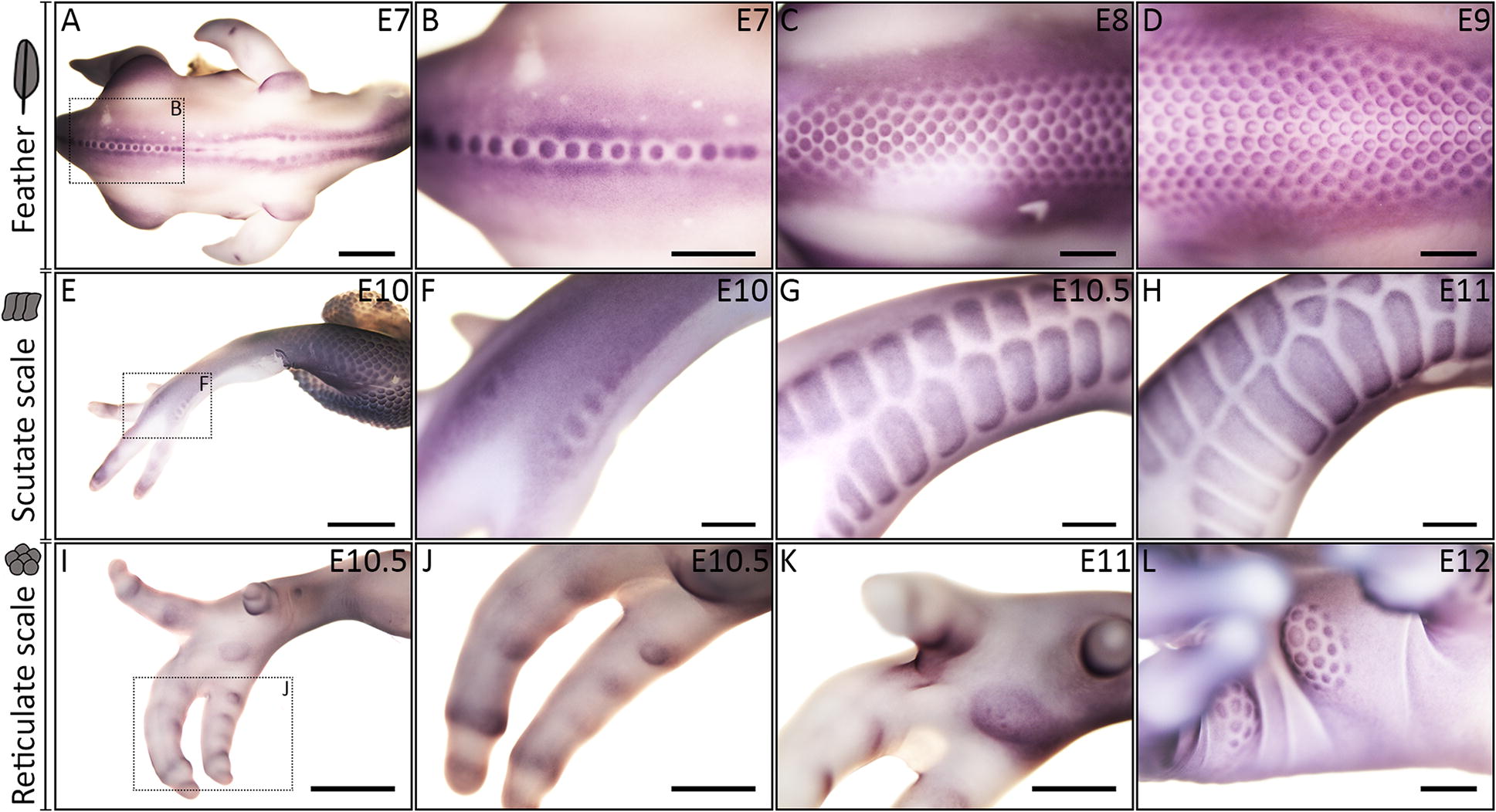
Localised β-catenin expression demarks feather, scutate and reticulate scale development. Whole-mount ISH for *β*-*cat* was performed to examine patterning of avian epithelial appendages. Feather patterning begins at E7, with a bifurcating dorsolateral row of feathers developing within an initiatory tract, triggering RD patterning of adjacent feathers [8] (**A**–**D**). Scutate scales form along the anterior metatarsal shank and dorsal foot surface, beginning at E10 (**E**–**H**). Restricted circular domains of *β*-*cat* preceding individual reticulate scales are visible at E10.5 along the ventral surface of the footpad and digits (**I**–**K**). These domains appear to subsequently subdivide into smaller units at E12 (**L**), which then form individual reticulate scales. Scale bar lengths are as follows: **A**, **E** = 2000 µm, **B**, **C**, **D**, **I**, = 1000 µm, **J**, **K**, **L** = 500 µm, **F**, **G**, **H** = 400 µm

First, we aimed to investigate the diversity of both avian and squamate epithelial appendages. To do this, we used scanning electron microscopy (SEM) to examine morphological variations in the epithelial appendages of these evolutionarily distinct groups. Birds and squamates share a common ancestry within Diapsida, but their respective lineages diverged from each other approximately 255 million years ago [28]. Diverse feather types develop in tracts from the proximal–distal elongation of feather buds, covering most of the chicken embryo’s body (Fig. 1A). Scutate scales are large, overlapping, approximately rectangular structures found on the metatarsal shank and dorsal surface of the foot [20, 29]. Both feathers and scutate scales display anterior–posterior asymmetry (Fig. 1A, B) after developing from a radially symmetrical placode (Fig. 2A–P, Fig. 3A–H) [20]. Reticulate scales form on the ventral surface of the footpad and digits (Fig. 1C). Unlike feathers and scutate scales, they maintain radial symmetry in their adult form.

We next examined the morphology of squamate scales belonging to three lizard species, to discern the diversity of these structures. This included the veiled chameleon (*Chamaeleo calyptratus*) and the bearded dragon (*Pogona vitticeps*) which are members of Acrodonta, and the blue-headed anole (*Anolis allisoni*), which belongs to Pleurodonta [30]. Hatchling *C. calyptratus* possess bilateral overlapping scales on the dorsal surface of the feet (Fig. 1D). Scales on the ventral foot surface retain a similar shape to the dorsal scales, but do not overlap and appear thicker than those on the dorsal surface (Fig. 1D). These ventral foot scales are morphologically similar to chicken reticulate scales (Fig. 1C). Scales of hatchling *A. allisoni* are large, overlapping and approximately rectangular, with those on the ventral foot surface appearing comparable to chicken scutate scales, in terms of their general morphology (Fig. 1E). The scales of pre-hatchling (E46) *P. vitticeps* are similar to those of *A. allisoni*, as they are large, overlapping structures on both the dorsal and ventral foot surfaces (Fig. 1F).

Overall, there appears to be less morphological diversity between the scales present on ventral and dorsal foot surfaces of the lizard species examined here than observed in the chicken. Furthermore, we observed no clear boundary separating dorsal and ventral squamate scale types. Therefore, the scales on lizard dorsal and ventral foot surfaces may be modifications of a similar squamate scale morphology, whereas the chicken possesses morphologically distinct scale types: the scutate and reticulate scales [20].

### Conserved gene signalling is observed throughout the development of reticulate scales and other avian appendages

Next, we aimed to compare and understand the developmental pathways and mechanisms underlying the early formation of different avian epithelial appendages, including reticulate scales. Most epithelial appendages have been shown to develop from the initial formation of an anatomical placode, which arises within an initiatory field such as a feather tract [1, 5, 8]. The anatomical placode is defined by an epithelial thickening with columnar cells exhibiting a reduced rate of proliferation, along with conserved molecular signalling in both the epithelium and underlying mesenchyme [5]. First, to investigate cellular proliferation rate, we examined immunoreactivity of proliferating cell nuclear antigen (PCNA) during the early development of avian epithelial appendages (Fig. 2).

As shown previously, avian feathers and scutate scales both develop from anatomical placodes which first arise within initiatory fields at embryonic day 7 (E7) and E10, respectively [6, 8, 31]. These placodes exhibit columnar cells of the basal epithelium with a characteristically reduced rate of proliferation compared to surrounding cells [5] (Fig. 2A, I, white arrowheads). Notably, PCNA immunoreactivity indicated that reticulate scales first develop from comparatively larger epithelial thickenings that emerge along the ventral side of the footpad and digits at E10.5. These placodes also possess columnar basal epithelial cells with a slightly reduced proliferation compared to surrounding cells (Fig. 2Q, white arrowhead, Additional file 1: Figure S1).

We next aimed to investigate whether conserved molecular signalling in the epithelium and mesenchyme underlies the development of chicken epithelial appendages. First, we examined expression of the transcriptional cofactor β-catenin (*β*-*cat*), one of the earliest known epithelial regulators of primordium-specific gene expression [32] (Figs. 2, 3). Whole-mount ISH revealed *β*-*cat* demarcates the development of feathers, scutate and reticulate scales, from initiation through to morphogenesis (Fig. 3) [32, 33]. Whilst feather development involves anterior to posterior and lateral addition of primordia (Fig. 3A–D), similar to zebrafish scale patterning [34], scutate scale patterning occurs through the spread of placodes proximally along the metatarsal shank and distally along the digits (Fig. 3E–H). Some scutate scale placodes may fuse to produce enlarged scale buds [26]. Notably, localised expression of *β*-*cat* marks restricted circular domains along the ventral footpad and digits (E10.5, Fig. 3I–K), which appear to subsequently subdivide into individual reticulate scales (E12, Fig. 3L).

Sectioning of whole-mount ISH samples revealed that expression of *β*-*cat* was specific to the epithelium of developing feathers, scutate and reticulate scales, during both the primary epithelial thickening and morphogenesis stages (Fig. 2B, F, J, N, R, V). Additionally, we examined expression of a conserved regulator of epithelial appendage development, sonic hedgehog (*Shh*) [8, 35–37]. *Shh* expression was observed in the epithelium of developing appendages at both the placode and morphogenesis stages of development for feathers and scutate scales (Fig. 2C, G, K, O) [8]. Expression of *Shh* was not localised to the primary epithelial thickening stage of reticulate scales at E10.5, although we observed weak expression in the epithelium and underlying mesenchyme (Fig. 2S). During morphogenesis, expression of *Shh* was strong and specific to individual elevations of the epithelium (Fig. 2W). Finally, we charted the expression of bone morphogenetic protein 4 (*Bmp4*), a mesenchymal marker of placode development [5, 8]. *Bmp4* expression was limited to the mesenchyme during the primary epithelial thickening stage of feathers, scutate and reticulate scales (Fig. 2D, L, T), before also shifting to the epithelium during morphogenesis (Fig. 2H, P, X). We also observed localised expression of additional conserved markers including bone morphogenetic protein 2 (*Bmp2*) and sprouty 2 (*Spry2*) during reticulate scale development (Additional file 1: Figure S2). Together, these results demonstrate that conserved molecular signalling in both the epithelium and underlying mesenchyme regulates the early development of chick epithelial appendages, including reticulate scales.

Overall, these results support previous research suggesting that feathers and scutate scales develop from an anatomical placode [8, 36, 37]. This character is typified by columnar epithelial cells exhibiting a reduced rate of proliferation and conserved molecular signalling in both the epithelium and mesenchyme [5, 6, 32]. Additionally, we provide new developmental evidence that reticulate scales may develop following a similar system, initiating at E10.5.

### A derived patterning mechanism underlies chicken reticulate scale development

Previously, it has been suggested that reticulate scales do not develop from an anatomical placode but instead appear as symmetrical elevations at E12, although this event may be preceded by a placode spanning the entire foot or toe pad [6]. Here, we have provided evidence that circular domains of conserved localised gene expression arise upon the ventral surface of the footpad and digits before subsequent development of reticulate scales.

**Fig. 4:**
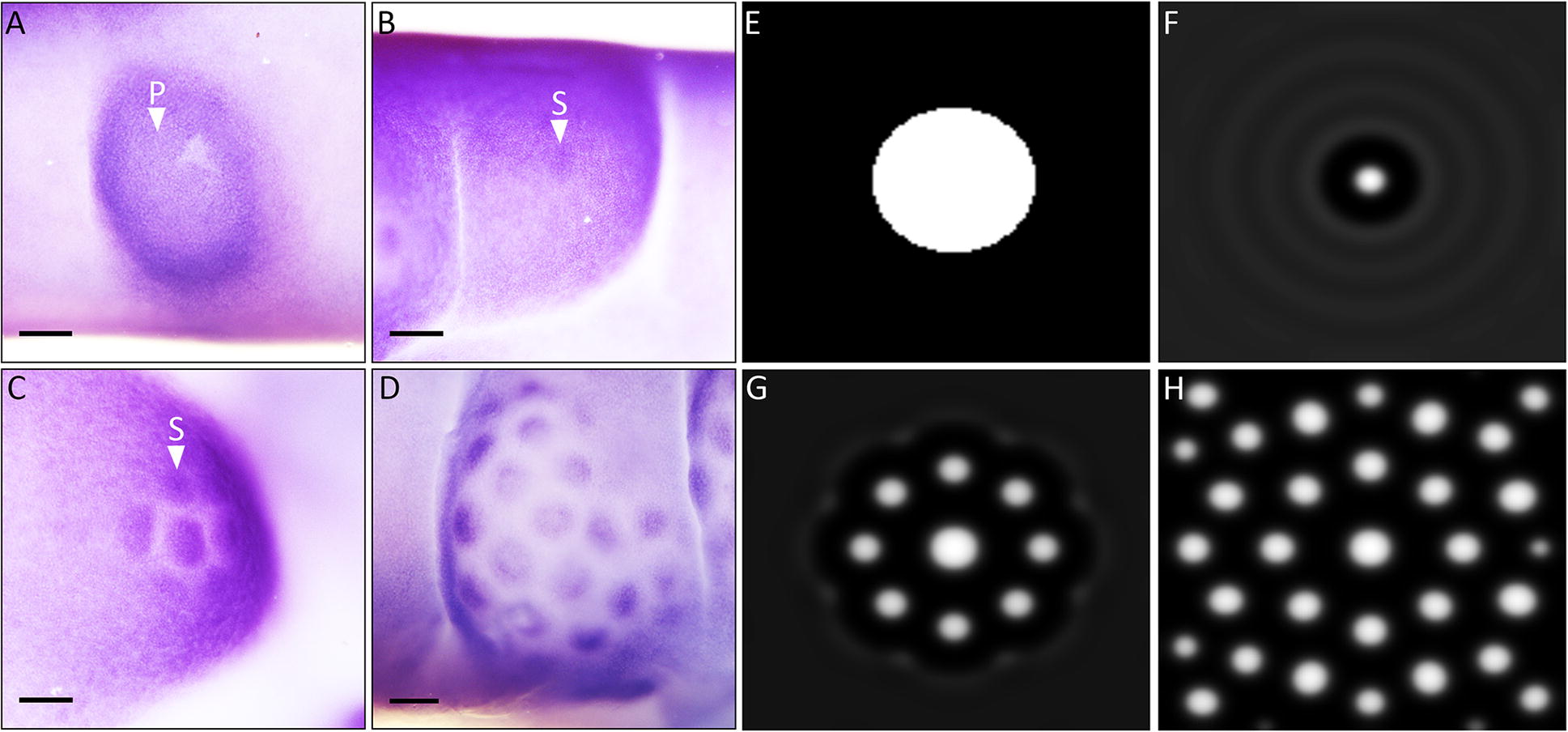
Reaction–diffusion simulation can explain the patterning of avian reticulate scales. Whole-mount ISH revealed that reticulate scale development begins with a circular domain (**A**, white arrowhead P), which subsequently subdivides into smaller secondary units, radiating outwards sequentially out from a central unit (**B**–**D**, white arrowhead S). RD simulation suggests that interactions between diffusing activatory and inhibitory morphogens can explain this patterning process (**E**–**H**). See “Methods” section for further details of RD modelling. Scale bars are 250 µm in length

The epithelial thickenings that subsequently give rise to reticulate scales emerge along the digits at E10.5 (Figs. 2Q–T, 3I–L). These circular domains are larger than the initial placodes that give rise to feathers and scutate scales, and appear to subdivide into smaller, secondary units, which radiate outwards sequentially from a central unit (Fig. 4A–D). They subsequently undergo morphogenesis to become radially symmetrical reticulate scales (Fig. 1C). Such periodic patterning bears striking similarity to a RD system, similar to that which underlies avian feather patterning [8]. Feather patterning involves a bifurcating dorsolateral initiator row of placodes triggering the emergence of parallel, adjacent rows [8]. During reticulate scale patterning, we observed enlarged placode-shaped domains, which appear to subdivide into radially arranged smaller secondary units, as opposed to the emergence of placodes in parallel, adjacent rows in feather development [8] (Fig. 3I–L). Reticulate scale patterning may follow a derived RD mechanism, adapted from the system that underpins feather or scutate scale development.

Diverse vertebrate epithelial appendages are thought to be patterned through RD, in which interactions between diffusing activatory and inhibitory morphogens result in autonomous pattern formation [8, 13, 14]. Therefore, we examined whether RD simulation can explain the propagation of reticulate scales from a single, circular initiatory domain (Fig. 4E–H). We initialised a RD simulation with a central spot representing the primary epithelial thickening (Fig. 4E). Numerical exploration revealed a range of model parameter values for which waves of activatory and inhibitory signals radiated from the primary placode (Fig. 4E–H, see “Methods” for further details). From this simulation, we observed the enlarged primary domain subdividing into smaller secondary units, added sequentially from a central unit in a radial arrangement (Fig. 4E–H). This is comparable to expression patterns of *β*-*cat* observed from E10.5 to E12 (Fig. 4A–D). These results demonstrate that RD can theoretically explain the derived patterning mechanism underpinning the development of reticulate scales.

**Fig. 5:**
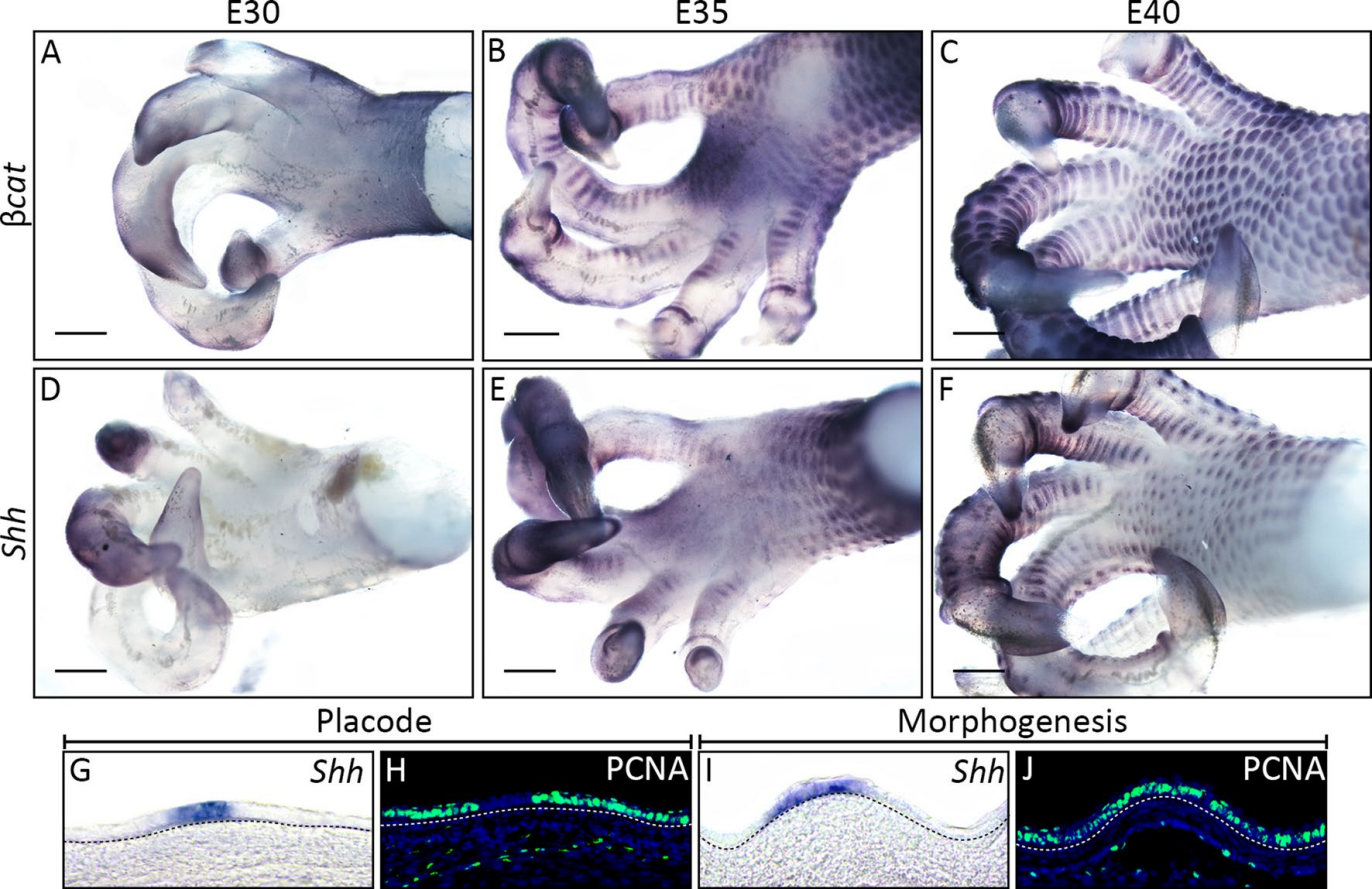
Scales of the bearded dragon ventral footpad arise synchronously from individual placodes. Whole-mount ISH was performed to investigate gene expression during scale development of the bearded dragon’s (*P. vitticeps)* ventral foot surface. At E30, no placodes were visible (**A**, **D**). By E35, placodes were visible emerging synchronously over the footpad and digits, expressing both *β*-*cat* and *Shh* (**B**, **E**). By E40, these units had developed to cover the footpad and digits, still expressing *β*-*cat* and *Shh* (**C**, **F**). Section ISH of bearded dragon body scales revealed that *Shh* expression is epithelial during both placode stage and morphogenesis (**G**, **I**), as previously described [5]. PCNA immunoreactivity revealed that columnar cells of the basal epithelium exhibit a reduced rate of proliferation in the placode stage (**H**), compared to morphogenesis (**J**). Dashed lines separate the basal epithelium from the underlying mesenchyme. Scale bars are 500 µm in length

Squamates also possess distinct epithelial appendages on the ventral surfaces of their feet. This observation, in combination with the presence of reticulate scales in birds, led to the suggestion that the ancestral archosaur would have also possessed distinct reticulate scales [25]. To test this hypothesis, we examined scale development on the ventral footpad of a reptilian squamate, the bearded dragon (*P. vitticeps*) (Fig. 5A–J). Reptilian body scales are known to develop from anatomical placodes [5] (Fig. 5G–J). ISH of *P. vitticeps* samples revealed that scales of the ventral footpad and digits also develop from individual placodes that begin to emerge synchronously at E35, and express both *Shh* and *β*-*cat* (Fig. 5A–F). Therefore, the footpad scales of *P. vitticeps* are developmentally distinct from avian reticulate scales in terms of their patterning, as reticulate scales arise from restricted, circular domains which subdivide into individual units (Figs. 2, 3, 4). This provides evidence that reticulate scales are derived epithelial appendages that are not present in squamates, at least in the bearded dragon, rendering the condition in the ancestral archosaur ambiguous.

## Discussion

Overall, we provide evidence that conserved gene signalling underlies the development of avian reticulate scales. Restricted, circular domains of conserved localised gene expression appear along the ventral footpad surface at E10.5. These domains appear to subdivide into individual radially arranged reticulate scales by E12, following a pattern consistent with RD simulation.

One important question that remains is whether this primary initiatory domain constitutes an enlarged anatomical placode or an initiatory field, comparable to the avian feather tract. Anatomical placodes are characterised by conserved gene expression in the epithelium and underlying mesenchyme, and a local epithelial reduction in cell proliferation [5]. We show some evidence for this in avian reticulate scales (Figs. 2Q–X, 3I–J, Additional file 1: Figure S2), although we did not observe localised expression of *Shh*, a widely conserved marker of skin appendage development, in the primary circular domain [38]. Therefore, it remains uncertain whether these circular domains are anatomical placodes, or a series of initiatory fields. Comparative transcriptome analysis of this primary circular domain with both feather tracts and placodes would help to resolve this question.

Our results demonstrate that the patterning of reticulate scales from an initial circular domain can be explained through RD simulation. RD controls the patterning of various vertebrate epithelial appendages [8, 14], and alterations to this system can give rise to diverse patterns both within and between different species, facilitating important functional traits [13]. We propose that reticulate scale patterning may follow a modified RD system, derived from the patterning of feathers or scutate scales. Although the patterning of reticulate scales appears distinct from the patterning of other avian epithelial appendages, it is likely still underpinned by a RD system.

It has been suggested that squamate scales are more similar to avian reticulate scales than feathers or scutate scales [22]. However, our developmental findings support the hypothesis that reticulate scales are derived structures [5], thus suggesting a new evolutionary relationship between different squamate and avian scale types. Fossil evidence has revealed that structures comparable to feathers, scutate and reticulate scales were present in coelurosaurian theropods [39, 40], although the prevalence of feathers in other dinosaur groups remains controversial [41–43]. Scale impressions are known for ornithischian and sauropodomorph dinosaurs, from both footprints and body fossils, but on the basis of the available morphological evidence it is currently ambiguous whether these were developmentally homologous with those of squamates or birds. However, one recent phylogenetic analysis of dinosaur evolution suggested that ornithischians and theropods share a sister group relationship, forming the clade Ornithoscelida [44]. If correct, this hypothesis might increase the likelihood that ornithischian ‘feathers’ and scales, which have been suggested to include both scutate and reticulate scales [41], were homologous with those of theropods as these could have been features present in the ornithoscelidan ancestor [41, 43, 44] (Additional file 1: Figure S3). Consequently, current evidence supports the appearance of reticulate scales early in theropod evolution [39], prior to the origin of birds, and it is plausible that they are an even more ancient dinosaurian feature.

Recent RNA-seq analysis of avian epithelial appendage types has indicated that feathers and scutate scales are more similar to each other, and to alligator scale types, than reticulate scales [25]. Researchers proposed that reticulate scales may have therefore arisen relatively earlier in tetrapod evolution. However, our results demonstrate that reticulate scales develop from restricted circular domains at E10.5, which may constitute an anatomical placode. Prior research has suggested that reticulate scales emerge as symmetrical elevations at E12 [6]. Therefore, this analysis may not have compared true placode stages between epithelial appendage types, providing an explanation for this dissimilarity. Additionally, this previous study showed that gene expression of scutate scales clustered with that of reticulate scales during morphogenesis [25], which is indicative of their developmental similarity in later development. Reticulate scales may be more developmentally similar to other avian appendage types than previously thought, as it is possible that they develop from an anatomical placode.

There is a degree of morphological similarity between squamate scales of the veiled chameleon (*C. calyptratus*) and avian reticulate scales (Fig. 1C, D). However, based on the development of these units we propose this similarity is a result of convergent evolution, with scales on the ventral foot surfaces of both groups having evolved to fulfil similar functions, such as grip and cushioning [20, 45]. Despite their similarity in appearance, reptilian ventral footpad scales are developmentally distinct from reticulate scales, as their patterning follows the synchronous emergence of individual placodes at E35, rather than the subdivision of a circular domain (Figs. 4, 5).

## Conclusion

Overall, we demonstrate that the development of avian epithelial appendages, including feathers, scutate and reticulate scales, is regulated by the signalling of conserved developmental genes. During reticulate scale development, circular domains of localised gene expression are observed along the ventral footpad at E10.5, constituting either anatomical placodes or circular initiatory fields. These domains subsequently subdivide into individual reticulate scales, following a patterning mechanism consistent with RD simulation. This is distinct from the patterning of squamate (*P. vitticeps*) ventral footpad scales. Therefore, we suggest that reticulate scales are derived epithelial appendages patterned through a modified RD system.

## Methods

### Animal husbandry

The University of Sheffield is a licensed establishment under the Animals (Scientific Procedures) Act 1986. All animals were culled by approved methods cited under Schedule 1 to the Act. Fertilised chicken eggs (Bovan Brown, Henry Stewart & Co., Norfolk, UK) were incubated at 37.5 °C and fixed overnight in Carnoy’s solution. Embryos were dehydrated into ethanol (EtOH) and stored at − 20 °C. *A. allisoni* and *C. calyptratus* specimens were a gift from Oldřich Zahradníček. *P. vitticeps* embryos were obtained from reptile breeding facility at the University of Helsinki (licence ESAVI/13139/04.10.05/2017).

### Scanning electron microscopy (SEM)

SEM was performed using a Hitachi TM3030Plus Benchtop SEM scanning at 15,000 V. Samples were rehydrated to PBS, washed in ddH20 and air-dried before scanning.

### Haematoxylin and eosin (H&E) staining

H&E staining was performed as previously described [7]. Imaging was carried out using an Olympus BX51 microscope and Olympus DP71 Universal digital camera attachment.

### In situ hybridisation (ISH)

Whole-mount ISH was performed as previously described [7], using riboprobes synthesised from the Riboprobe System Sp6/T7 kit (Promega) and DIG labelling mix (Roche). Primer sequences are as follows: Chick *β*-*cat* (forward: TCTCACATCACCGTGAAGGC, reverse: CCTGATGTCTGCTGGTGAGG). Data obtained from plasmids used to synthesise bearded dragon *β*-*cat* and *Shh*, and chick *Spry2*, *Shh*, *Bmp2* and *Bmp4*, have previously been published [5, 46–48]. A minimum of 6 samples were used for ISH for each gene at each stage of chicken development. As bearded dragon embryos were comparatively scarce, 3 samples were used per gene at each developmental stage. Samples were imaged using a Nikon SMZ15000 stereomicroscope. Vibratome sections were cut at a thickness of 30 µm and imaged using an Olympus BX51 microscope and Olympus DP71 universal digital camera attachment. Brightness and contrast were adjusted to improve clarity. Scale bars were added using Fiji [49]. Cryosections after whole-mount ISH in bearded dragon were performed as previously described [5].

### Immunofluorescence

Immunofluorescence for PCNA was done as previously described [5, 7]. Imaging was carried out with an Olympus BX61 upright epifluorescent microscope and Olympus DP71 universal digital camera attachment, using the software Volocity 6.3.

### Reaction–diffusion (RD) modelling

RD modelling of reticulate scale patterning was done using an activator–inhibitor model proposed by Kondo and Miura [12], as previously described [13]. Briefly, this model describes the diffusion of, and nonlinear reaction between, activator (*u*) and inhibitor (*v*) molecules in a two-dimensional domain. Parameter values were as follows: $d_{u}$ = 0.02, $D_{u}$ = 0.02, $a_{u}$ = 0.06, $b_{u}$ = − 0.07, $c_{u}$ =0.015, $F_{ \text{max} }$ = 0.19, $d_{v}$ = 0.031, $D_{v}$ = 0.4, $a_{v}$ = 0.0608, $b_{v}$ = 0.004, $c_{v}$ = − 0.025, $G_{ \hbox{max} } = 0.184$. For the simulations shown in Fig. 4E–H, we specified the initial condition

1 $$u\left( {0,x,y} \right)\left\{ \begin{aligned} & u_{0} \quad {\text{if }}\left( {x - L/2} \right)^{2} + \left( {y - L/2} \right)^{2} < R^{2} , \\ & 0\quad \;{\text{otherwise}}, \\ \end{aligned} \right.$$

2 $$v\left( {0,x,y} \right) = 0,$$

defined in a square spatial domain $0 < x,y < L$ with no-flux boundary conditions. Parameter values used were $L = 75$ and $L = 1.5$. This central ‘spot’ represents a primary reticulate placode. These values were determined based on an ad hoc exploration around values previously shown to result in patterning [12]. See Cooper et al. [13] for further details of reaction–diffusion modelling.

## Additional file

**Additional file 1.** Additional figures.

**Additional file 2.** Python script for reaction-diffusion simulations.

## Data Availability

The dataset supporting the conclusions of this article is included within the article. The Python code used to simulate RD pattern formation in Fig. 4 is included in the Additional file 2.

## Abbreviations

RD: reaction–diffusion
SEM: scanning electron microscopy
ISH: in situ hybridisation
PCNA: proliferating cell nuclear antigen
*β*-*cat*: β-catenin (gene)
*Shh*: sonic hedgehog (gene)
*Bmp4*: bone morphogenetic protein 4 (gene)
*Bmp2*: bone morphogenetic protein 2 (gene)
*Spry2*: sprouty 2 (gene)

## References

[1] Pispa J, Thesleff I (2003). Mechanisms of ectodermal organogenesis. Dev Biol.

[2] Reif W-E (1985). Functions of Scales and Photophores in mesopelagic luminescent sharks. Acta Zool..

[3] Ruxton GD, Wilkinson DM (2011). Avoidance of overheating and selection for both hair loss and bipedality in hominins. Proc Natl Acad Sci.

[4] Dean B, Bhushan B (2010). Shark-skin surfaces for fluid-drag reduction in turbulent flow: a review. Philos Trans A Math Phys Eng Sci.

[5] Di-Poï N, Milinkovitch MC (2016). The anatomical placode in reptile scale morphogenesis indicates shared ancestry among skin appendages in amniotes. Sci Adv.

[6] Musser JM, Wagner GP, Prum RO (2015). Nuclear β-catenin localization supports homology of feathers, avian scutate scales, and alligator scales in early development. Evol Dev.

[7] Cooper RL, Martin KJ, Rasch LJ, Fraser GJ (2017). Developing an ancient epithelial appendage: FGF signalling regulates early tail denticle formation in sharks. Evodevo.

[8] Jung H (1998). Local inhibitory action of BMPs and their relationships with activators in feather formation: implications for periodic patterning. Dev. Biol..

[9] Tanaka S, Kato Y (1983). Epigenesis in developing avian scales. II. Cell proliferation in relation to morphogenesis and differentiation in the epidermis. J Exp Zool.

[10] Ahtiainen L (2014). Directional cell migration, but not proliferation, drives hair placode morphogenesis. Dev Cell.

[11] Turing AM (1952). The chemical basis of morphogenesis. Philos Trans R Soc Lond B Biol Sci.

[12] Kondo S, Miura T (2010). Reaction-diffusion model as a framework of understanding biological pattern formation. Science.

[13] Cooper RL (2018). An ancient Turing-like patterning mechanism regulates skin denticle development in sharks. Sci Adv.

[14] Sick S, Reinker S, Timmer J, Schlake T (2006). WNT and DKK determine hair follicle spacing through a reaction-diffusion mechanism. Science.

[15] Milinkovitch MC (2013). Crocodile head scales are not developmental units but emerge from physical cracking. Science.

[16] Shyer AE (2017). Emergent cellular self-organization and mechanosensation initiate follicle pattern in the avian skin. Science.

[17] Ho WKW (2019). Feather arrays are patterned by interacting signalling and cell density waves. PLoS Biol.

[18] Prum RO (1999). Development and evolutionary origin of feathers. J Exp Zool.

[19] Stettenheim PR (2000). The integumentary morphology of modern birds—an overview. Amer. Zool..

[20] Chuong C-M, Chodankar R, Widelitz RB, Ting-Xin J (2000). Evo-devo of feathers and scales: building complex epithelial appendages. Curr Opin Genet Dev.

[21] Dhouailly D (2009). A new scenario for the evolutionary origin of hair, feather, and avian scales. J Anat.

[22] Sawyer RH, Knapp LW, Guin WM, Bereiter-Hahn J, Matoltsy AG, Richards KS (1986). Epidermis, dermis and appendages. Biology of the integument.

[23] Brush AH, Wyld JA (1980). Molecular correlates of morphological differentiation: avian scutes and scales. J Exp Zool.

[24] Sawyer RH, Craig KF (1977). Avian scale development absence of an “epidermal placode” in reticulate scale morphogenesis. J Morphol.

[25] Musser JM (2018). Subdivision of ancestral scale genetic program underlies origin of feathers and avian scutate scales. Biorxiv..

[26] Wu P, Lai Y-C, Widelitz R, Chuong C-M (2018). Comprehensive molecular and cellular studies suggest avian scutate scales are secondarily derived from feathers, and more distant from reptilian scales. Sci Rep.

[27] Harris MP, Williamson S, Fallon JF, Meinhardt H, Prum RO (2005). Molecular evidence for an activator-inhibitor mechanism in development of embryonic feather branching. Proc Natl Acad Sci.

[28] Brusatte SL, O’Connor JK, Jarvis ED (2015). The origin and diversification of birds. Curr Biol.

[29] Sawyer RH (1972). Avian scale development: histogenesis and morphogenesis of the epidermis and dermis during formation of the scale ridge. J Exp Zool.

[30] Wiens JJ (2012). Resolving the phylogeny of lizards and snakes (Squamata) with extensive sampling of genes and species. Biol Lett.

[31] Harris MP, Fallon JF, Prum RO (2002). Shh-Bmp2 signaling module and the evolutionary origin and diversification of feathers. J Exp Zool.

[32] Noramly S, Freeman A, Morgan BA (1999). β-catenin signaling can initiate feather bud development. Development.

[33] Widelitz RB, Jiang TX, Lu J, Chuong CM (2000). Beta-catenin in epithelial morphogenesis: conversion of part of avian foot scales into feather buds with a mutated beta-catenin. Dev Biol.

[34] Aman AJ, Fulbright AN, Parichy DM (2018). Wnt/β-catenin regulates an ancient signaling network during zebrafish scale development. Elife.

[35] Chiang C (1999). Essential role for sonic hedgehog during hair follicle morphogenesis. Dev Biol.

[36] Ting-berreth SA, Chuong C (1996). Sonic hedgehog in feather morphogenesis : induction of mesenchymal condensation and association with cell death. Dev Dyn.

[37] Morgan BA, Orkin RW, Noramly S, Perez A (1998). Stage-specific effects of sonic hedgehog expression in the epidermis. Dev Biol.

[38] Chuong CM, Patel N, Lin J, Jung HS, Widelitz RB (2000). Sonic hedgehog signaling pathway in vertebrate epithelial appendage morphogenesis: perspectives in development and evolution. Cell Mol Life Sci.

[39] Cuesta E, Díaz-Martínez I, Ortega F, Sanz JL (2015). Did all theropods have chicken-like feet? First evidence of a non-avian dinosaur podotheca. Cretac Res.

[40] Fucheng Z, Zhonghe Z, Dyke G (2006). Feathers and ‘feather-like’ integumentary structures in Liaoning birds and dinosaurs. Geol J.

[41] Godefroit P (2014). A Jurassic ornithischian dinosaur from Siberia with both feathers and scales. Science.

[42] Barrett PM, Evans DC, Campione NE (2015). Evolution of dinosaur epidermal structures. Biol Lett.

[43] Yang Z (2019). Pterosaur integumentary structures with complex feather-like branching. Nat Ecol Evol.

[44] Baron MG, Norman DB, Barrett PM (2017). A new hypothesis of dinosaur relationships and early dinosaur evolution. Nature.

[45] Chang C (2009). Reptile scale paradigm: evo-devo, pattern formation and regeneration. Int J Dev Biol.

[46] Chambers D, Mason I (2000). Expression of sprouty2 during early development of the chick embryo is coincident with known sites of FGF signalling. Mech Dev.

[47] Riddle RD, Johnson RL, Laufer E, Tabin C (1993). Sonic hedgehog mediates the polarizing activity of the ZPA. Cell.

[48] Pickering J, Wali N, Towers M (2017). Transcriptional changes in chick wing bud polarization induced by retinoic acid. Dev Dyn.

[49] Schindelin J (2012). Fiji: an open-source platform for biological-image analysis. Nat Methods.

