# Supplementary material for: Conserved gene signalling and a derived patterning mechanism underlie the development of avian footpad scales
Source: EvoDevo. 2019 Aug 13;10:19. doi: 10.1186/s13227-019-0130-9 (PMC6693258; doi:10.1186/s13227-019-0130-9)
Supplement: Supplementary file 1 — Additional file 1. Additional figures. [file 13227_2019_130_MOESM1_ESM.docx]

Additional information for Cooper *et al.* 2019:


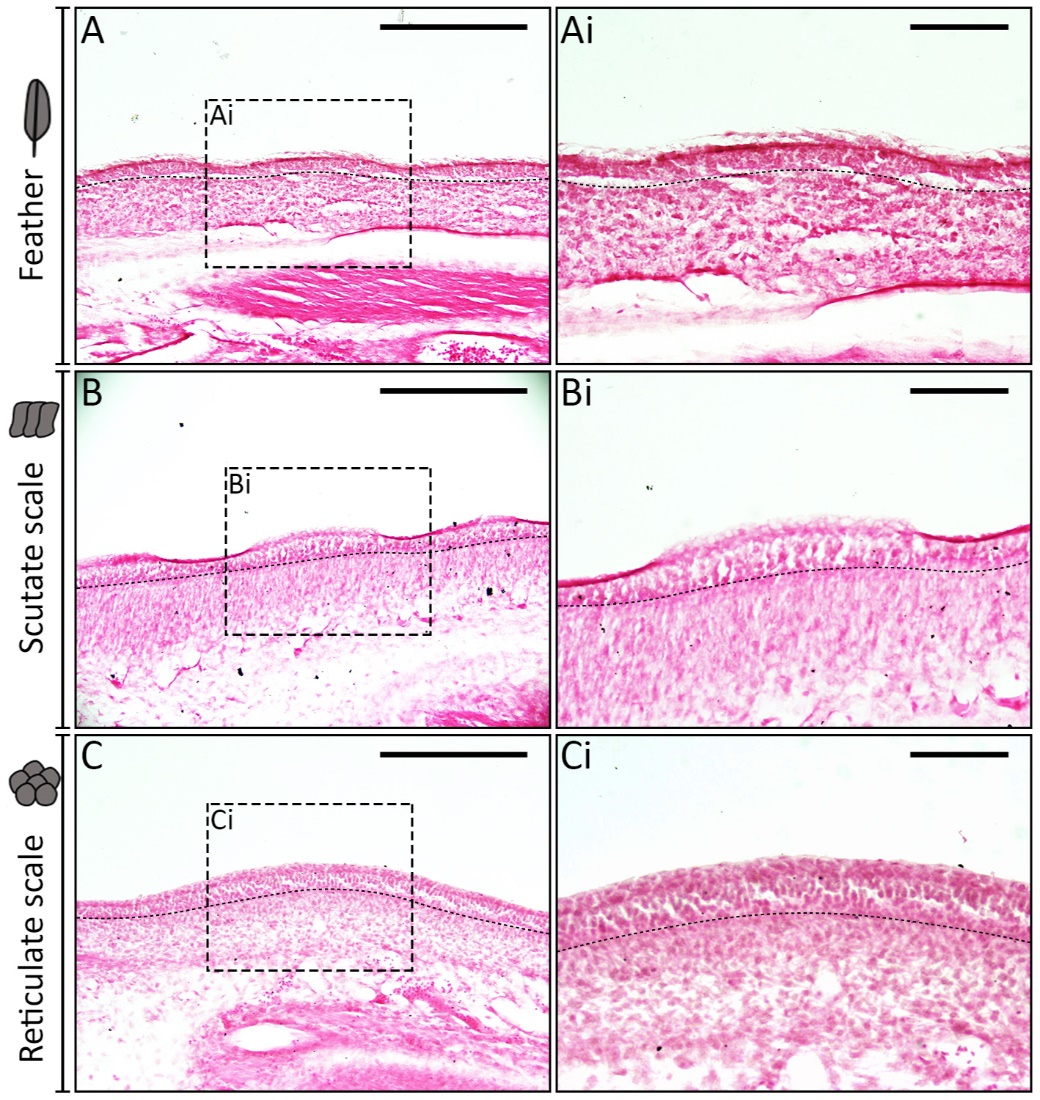
Conserved gene signalling and a derived patterning mechanism underlie the development of avian footpad scales

**Additional Figure 1: Haematoxylin and eosin staining of chick appendage placodes.** Paraffin embedded E10.5 chicken embryos were microtome sectioned and stained with haematoxylin and eosin. As previously shown^6^, feathers and scutate scales develop from a placode with localised columnar epithelial cells (A, B). Additionally, we observed localised thickening of columnar basal epithelial cells specific to early reticulate scale development (C). Black dashed lines separate the basal epithelium from the mesenchyme. Scale bar lengths are: A, B, C =150µm, and Ai, Bi, Ci = 75µm.


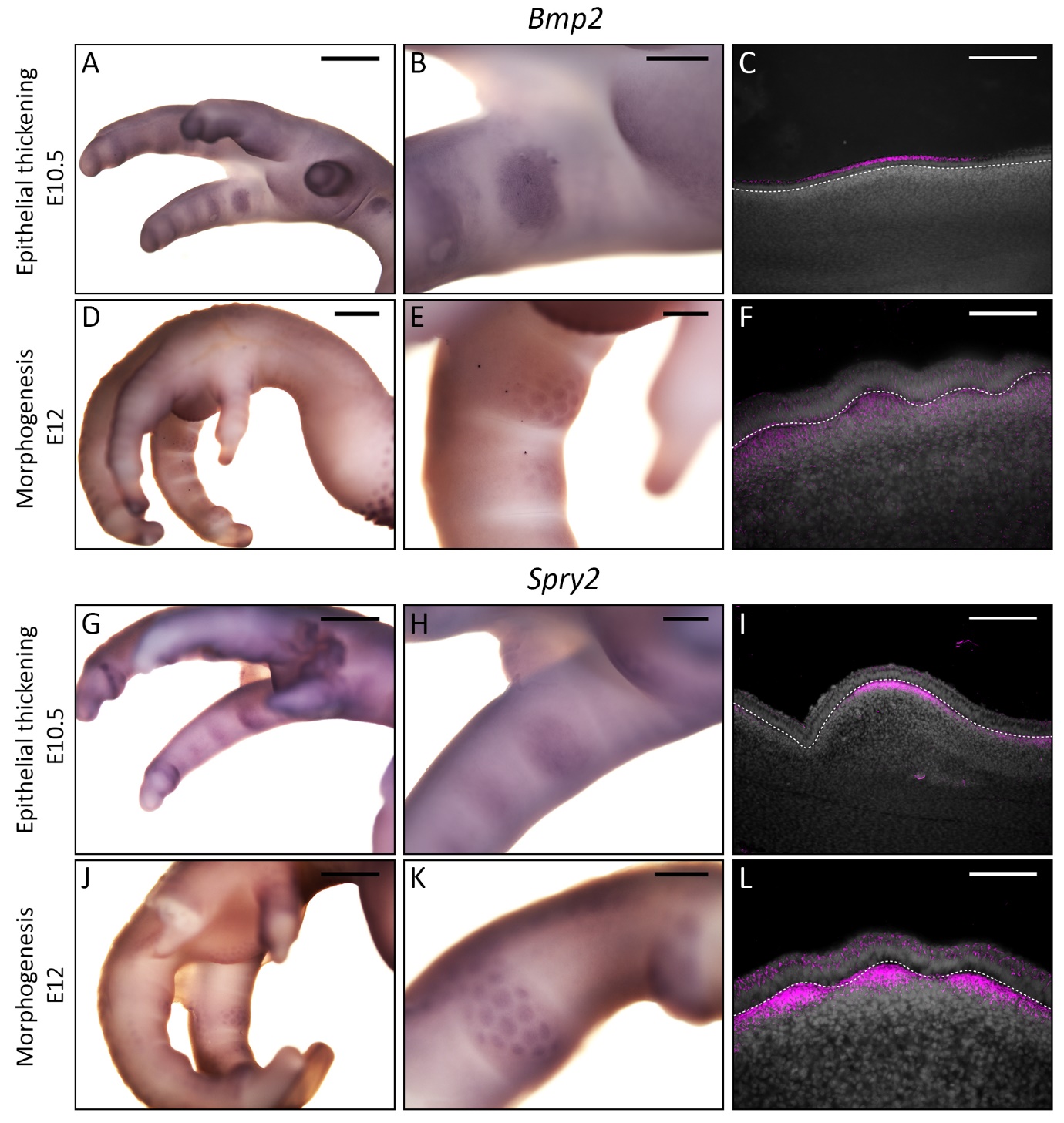


**Additional Figure 2: Additional conserved gene expression during reticulate scale development.** Similarly to feather development^28^, we observed expression of *Bmp2* in the epithelial thickening during chick reticulate placode development (E10.5) (A-C), and the mesenchyme during morphogenesis (E12) (D-F). Furthermore, we observed mesenchymal expression of *Spry2*, a regulator of fibroblast growth factor signalling^48,49^. In the epithelial thickening stage (E10.5) (G-I), *Spry2* expression was mesenchymal, and during morphogenesis weak expression was also observed in the epithelium (E12) (J-L). White dashed lines separate the basal epithelium from the underlying mesenchyme. Scale bar lengths are as follows: A, D, G, J = 1000µm, B, E, H, K = 300µm, C, I = 150µm, F, L = 75µm.


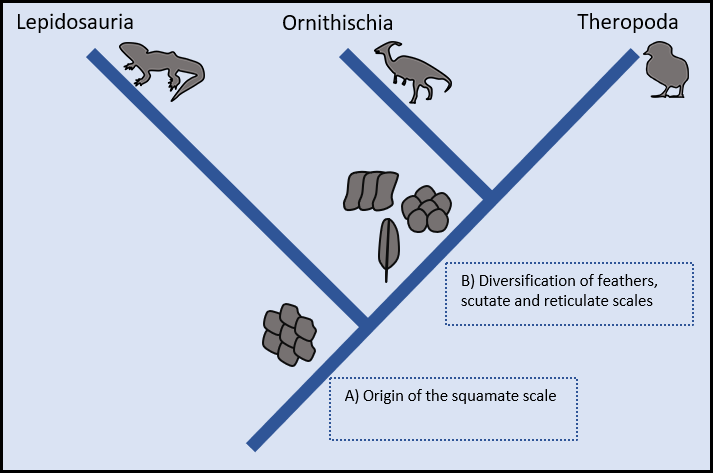


**Additional Figure 3: A hypothesis for the evolutionary relationships between squamate and avian appendage types.** We propose that feathers, scutate scales and reticulate scales diversified after the divergence of Lepidosauria (which includes squamates), and prior to the divergence of Ornithischia, as they have been observed in both ornithischian and theropod fossils^37,39^. These structures are retained in extant avian species, which arose from theropod dinosaurs^47^.
